# A Comparative Assessment of Cardiovascular Autonomic Reflex Testing and Cardiac ^123^I-Metaiodobenzylguanidine Imaging in Patients with Type 1 Diabetes Mellitus without Complications or Cardiovascular Risk Factors

**DOI:** 10.1155/2018/5607208

**Published:** 2018-03-12

**Authors:** Triantafyllos Didangelos, Efstratios Moralidis, Eleni Karlafti, Konstantinos Tziomalos, Charalambos Margaritidis, Zisis Kontoninas, Ioannis Stergiou, Maria Boulbou, Marianthi Papagianni, Emmanouel Papanastasiou, Apostolos I. Hatzitolios

**Affiliations:** ^1^Diabetes Center, First Propedeutic Department of Internal Medicine, Medical School, Aristotle University of Thessaloniki, AHEPA Hospital, Thessaloniki, Greece; ^2^Laboratory of Nuclear Medicine, Medical School, Aristotle University of Thessaloniki, Papageorgiou Hospital, Thessaloniki, Greece; ^3^Department of Internal Medicine, Medical School, University of Thessaly, Larissa, Greece; ^4^Department of Medical Physics, Aristotle University of Thessaloniki, Thessaloniki, Greece

## Abstract

**Aim:**

To compare the cardiovascular autonomic reflex tests (CARTs) with cardiac sympathetic innervation imaging with ^123^I-metaiodobenzylguanidine (MIBG) in patients with type 1 diabetes mellitus (T1DM).

**Patients and Methods:**

Forty-nine patients (29 males, mean age 36 ± 10 years, mean T1DM duration 19 ± 6 years) without cardiovascular risk factors were prospectively enrolled. Participants were evaluated for autonomic dysfunction by assessing the mean circular resultant (MCR), Valsalva maneuver (Vals), postural index (PI), and orthostatic hypotension (OH). Within one month from the performance of these tests, patients underwent cardiac MIBG imaging and the ratio of the heart to upper mediastinum count density (H/M) at 4 hours postinjection was calculated (abnormal values, H/M < 1.80).

**Results:**

Twenty-nine patients (59%) had abnormal CARTs, and 37 (76%) patients had an H/M_4 < 1.80 (*p* = 0.456). MCR, PI, Vals, and OH were abnormal in 29 (59%), 8 (16%), 5 (10%), and 11 (22%) patients, respectively. When using H/M_4 < 1.80 as the reference standard, a cutoff point of ≥2 abnormal CARTs had a sensitivity of 100% but a specificity of only 33% for determining CAN.

**Conclusions:**

CARTs are not closely associated with ^123^I-MIBG measurements, which can detect autonomic dysfunction more efficiently than the former. In comparison to semiquantitative cardiac MIBG assessment, the recommended threshold of ≥2 abnormal CARTs to define cardiovascular autonomic dysfunction is highly sensitive but of limited specificity and is independently determined by the duration of T1DM.

## 1. Introduction

Diabetic neuropathy is the most common cause of neuropathy worldwide and the most common chronic complication of diabetes mellitus (DM) [1, 2]. Autonomic neuropathy is an underestimated complication of DM and may remain asymptomatic in early stages, especially in young patients with type 1 DM (T1DM) [3]. In the setting of DM, cardiovascular autonomic neuropathy (CAN) is defined as the impairment of the autonomic control of the cardiovascular system after the exclusion of other causes [2, 4]. CAN is one of the most serious consequences of DM due to its life-threatening complications, including silent myocardial infarction, ventricular arrhythmias, intraoperative cardiovascular instability, and sudden cardiac death, as well as because of its relation with other microvascular comorbidities [5, 6].

Over the years, much attention has been directed to the early diagnosis of CAN using standardized cardiovascular reflex tests (CARTs) [7]. These tests assess the parasympathetic system by evaluating beat-to-beat variations during deep breathing, moving from the supine to the standing position and during the Valsalva maneuver (Vals) [8]. On the other hand, orthostatic hypotension (OH) and the blood pressure response to Vals mostly evaluate the sympathetic system [8].

Nuclear imaging is also useful for the assessment of cardiac sympathetic innervation, by visualizing and measuring the uptake and storage of radiolabeled neurotransmitters into the presynaptic nerve terminals [9]. Several studies using ^123^I-metaiodobenzylguanidine (^123^I-MIBG) imaging have demonstrated the presence of abnormalities in sympathetic adrenergic innervation in diabetic patients [10–13].

It is noteworthy that most data on CAN are based on older studies, at a time when the treatment of diabetic patients might not have been optimal or the tests for identifying CAN might have been limited in number, performed in a nonstandardized manner, or influenced by comorbidities or medications [13, 14]. Moreover, the relation between CARTs and the more objective ^123^I-MIBG scintigraphy, in terms of direct visualization and quantification of cardiac sympathetic innervation, is not well determined [10, 15, 16].

The aim of the present study was to assess the CAN by both CARTs and ^123^I-MIBG scintigraphy in well-characterized T1DM patients, with disease duration > 5 years, without complications or cardiovascular risk factors, and who were only receiving insulin.

## 2. Patients and Methods

### 2.1. Patient Recruitment

Over an 18-month period, consecutive adult patients with T1DM who fulfilled the following criteria were enrolled in the study: (a) regular attendance of the outpatient diabetes clinic of our hospital to ensure systematic monitoring of glycemic control and diabetic complications, (b) diabetes duration > 5 years [17], (c) no symptoms associated with CAN (e.g., exercise intolerance, orthostatic intolerance, and syncope), (d) no clinically overt diabetic complications, other than CAN, or known cardiovascular risk factors and hence treated only with insulin, and (e) a normal adenosine myocardial perfusion scintigraphy. Ten patients had mild background retinopathy, and 6 patients had microalbuminuria. Thyroid stimulating hormone and thyroid hormones were measured in all patients because Hashimoto's thyroiditis is common in patients with T1DM. The levels of both thyroid stimulating hormone and thyroid hormones were within the normal range in all patients.

All patients had good glycemic control using insulin alone according to widely accepted standards of care [18]. Patients were well characterized and recruited for a broader study on diabetic autonomic dysfunction, which had been approved by the Ethics Committee of the Aristotle University of Thessaloniki. All patients provided informed consent.

### 2.2. Cardiovascular Autonomic Reflex Testing

Patients were submitted to a battery of CARTs early in the morning, as endorsed by international guidelines [7, 8, 19]. Subjects were instructed to abstain from caffeine or alcoholic beverages and smoking for a minimum of 8 h prior to testing and also from strenuous exercise for at least 24 h prior to testing. Patients with arrhythmias, fever, hypoglycemia, and emotional distress on the day before testing were excluded from testing. The following CARTs were performed:

1. The heart rate variation during deep breathing at 6 cycles per minute for 5 minutes, over 120 consecutive beats, was assessed with the mean circular resultant (MCR), which was measured by a vector analysis technique, as described elsewhere [18]. Patients, while lying down, were ordered to perform a deep and slow inspiration up to the maximum total lung capacity for 5 seconds, which was followed by a forced expiration down to the residual volume for 5 seconds. The time to alternate the respiratory cycle was signalled directly to the patient by the attending physician.
2. The postural index (PI) was calculated by measuring the heart rate response to the change of position from recumbent to standing over 180 seconds after getting up. This was calculated as the “30–15 ratio,” defined as the longest RR interval of beats 20–40 divided by the shortest RR interval of beats 5–25, starting from the first beat during the process of standing up.
3. For the assessment of heart rate variability during Vals, patients with the nose closed were asked to exhale into a mouthpiece connected to a manometer and to perform a continuous expiratory effort, which was equivalent to an intraoral pressure of 40 mmHg, for 15 seconds. The expiratory overstrain was then released, and patients were asked to breathe regularly until the end of the test. The ratio of the longest RR interval following the pressure release to the shortest RR interval during the maneuver was determined. The test was performed 3 times, and the mean value was used in the analyses.
4. For the OH test, blood pressure was measured with the patients in recumbent position, every minute for three minutes, and then while standing, every minute for 5 minutes. A drop of ≥20 mmHg in systolic blood pressure or ≥10 mmHg in diastolic blood pressure was considered abnormal.

Age-specific reference values were applied [20]. The first two CARTs address parasympathetic function, and Vals evaluates both parasympathetic and sympathetic function, whereas OH assesses sympathetic integrity.

### 2.3. ^123^I-MIBG Acquisition and Analysis

Within 1 month from performing the CARTs, patients were submitted to cardiac MIBG imaging. For this purpose, patients were treated with 130 mg potassium iodide orally for 3 days, starting on the day before tracer injection. There was no need to withhold medications interfering with MIBG imaging, since all patients were receiving only insulin [9]. 185 MBq of ^123^I-MIBG was administered slowly via a secured intravenous line in a peripheral vein.

Imaging was performed with a single-headed, large field of view ADAC Genesys SPECT gamma camera (ADAC Labs., Milpitas, CA, USA), equipped with a 3/8^″^ sodium iodide scintillation crystal and low-energy high-resolution collimator and interfaced with a Pegasys processing workstation. Anterior cardiac images were acquired at 15 minutes and 4 hours postinjection on a 256 × 256 matrix and an acquisition time of 600 sec, using a 15% energy window centered at the 159 keV photopeak of ^123^I.

The heart-to-mediastinum (H/M) count density ratio was calculated in a standardized manner, using an elliptical region of interest (ROI) placed over the heart and a rectangular ROI drawn over the upper mediastinum. Both the acquisition and measurement methodology are in line with official recommendations and have been previously validated in our institution [21–23]. For the purposes of this study, the H/M at 4 hours postinjection (H/M_4) was used in analysis, and the cutoff point of abnormal values was set at H/M_4 < 1.80, based on the published data [22, 24].

### 2.4. ^51^Cr-EDTA Glomerular Filtration Measurements

Within one month from the performance of CARTs, glomerular filtration rate (GFR) was measured in the morning, using the slope intercept and two sample technique, as described elsewhere [25]. In brief, blood samples were collected at 120 and 240 min after an intravenous injection of 3-4 MBq ^51^Cr-EDTA. A scintillation well counter (Cobra II, Packard, Meriden, CT, USA) was set to count plasma ^51^Cr, and the quadratic Brochner-Mortensen correction was applied in calculations [26]. GFR was measured with this technique because of the inaccuracy of various equations for the evaluation of GFR [27] and because CAN appears to predict the development of diabetic nephropathy [28].

### 2.5. Statistical Analysis

All data were analyzed with the statistical package SPSS (version 17.0; SPSS, Chicago, IL, USA). Data are presented as percentages for categorical variables and as mean and standard deviation for continuous variables. Differences between groups were assessed with paired or independent sample *t*-test, as appropriate, for continuous variables, and with chi-square test or Fisher's exact test, as appropriate, for categorical variables. One-way ANOVA was used when categorical or ordinal variables were compared between three or more groups, and Levene's test was used for evaluating the equality of variances. Stepwise backward binary logistic regression analysis was used to determine independent predictors, and a *p* value < 0.20 in univariate analysis was required for a variable to enter the multivariate model. A value of *p* < 0.05 was used to define statistical significance.

## 3. Results

Forty-nine patients with T1DM were enrolled in the study (29 males, mean age 36 ± 10 years (range, 19–62 years), mean DM duration 19 ± 6 years (range, 7–31 years)). Patients' characteristics are shown in Table 1. HbA_1c_ was also measured within 1 month from CARTs. Blood samples from 37 patients were analyzed in our institution, whereas the rest were tested elsewhere because of temporary logistic constraints. As deviations in the latter HbA_1c_ measurements were strongly suspected subsequently, only results from our laboratory were taken into account in this study, which are presented in a supplementary analysis. Similarly, ^51^Cr-EDTA GFR measurements within 1 month from CARTs were available for 46 patients, and thus renal function is analyzed separately, together with the 37 patients in whom HbA_1c_ was measured in our institution.

Twenty-nine patients (59%) had abnormal CARTs, and 37 patients (76%) had an H/M_4 < 1.80 (*p* = 0.456). MCR, PI, Vals, and OH were abnormal in 29 (59%), 8 (16%), 5 (10%), and 11 (22%) patients, respectively. The MIBG assessment of CAN as a function of the number of abnormal CARTs is presented in Table 2. Among the 36 patients with 0 or 1 abnormal CARTs, 24 patients (67%) also had sympathetic impairment in cardiac MIBG scintigraphy.

All 16 patients with only 1 abnormal CART had abnormal MCR. Abnormality in any one of the remaining CARTs was invariably accompanied by at least a second abnormal test. There were also 11 patients with abnormal OH test (H/M_4 = 1.50 ± 0.14), which were accompanied by 1 additional abnormal CART in 4 patients (H/M_4 = 1.45 ± 0.20) and 2 or more additional abnormal CARTs in 7 patients (H/M_4 = 1.53 ± 0.10). There was no significant difference between this latter group of 7 patients and the rest of the patients (1.66 ± 0.21, *p* = 0.115). When a more stringent cutoff point was used to assess advanced CAN, 5/7 (71%) patients with OH had an H/M_4 < 1.60 versus 19/42 patients (45%, *p* = 0.247).

In Table 3, the H/M_4 ratio is compared between patients divided according to the number of abnormal CARTs. Based on cardiac ^123^I-MIBG, a threshold of ≥2 abnormal CARTs could determine significant differences in the assessment of CAN with cardiac MIBG imaging. When using H/M_4 < 1.80 as a reference standard, a cutoff point of ≥2 abnormal CARTs had a sensitivity of 100% but a specificity of 33% in determining CAN.

There was no difference between males and females in mean age (35 ± 8 versus 37 ± 11 years, resp.; *p* = 0.356), mean DM duration (18 ± 6 versus 19 ± 7 years, resp.; *p* = 0.520), number of abnormal CARTs (*p* = 0.335), H/M_4 (1.67 ± 0.23 versus 1.60 ± 0.17, resp.; *p* = 0.296), and ^51^Cr-EDTA GFR (102 ± 18 versus 93 ± 15, resp.; *p* = 0.086), but males had lower HbA_1c_ than females (6.5% ± 1.4 versus 7.7% ± 2.0, resp.; *p* = 0.038).

**Figure 1:**
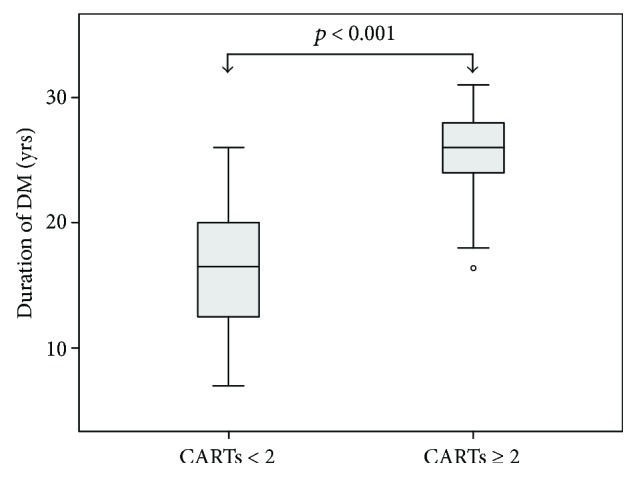
Box plots of the duration of type 1 diabetes mellitus in patients divided according to a cutoff point of ≥2 abnormal cardiovascular autonomic reflex tests (CARTs).

Study participants with ≥2 abnormal CARTs were older (42 ± 11 versus 33 ± 8 years, *p* = 0.003) and had longer DM duration (25 ± 5 versus 16 ± 5 years, *p* < 0.001) than patients with <2 abnormal CARTs (Figure 1). In binary logistic regression analysis, only DM duration was a significant predictor of ≥2 abnormal CARTs in the entire study population (model's chi-square 21.010, *p* < 0.001).

In the 37 patients with available HbA_1c_ measurements, there were significant differences between those with ≥2 abnormal CARTs and those with 0-1 abnormal CARTs in HbA_1c_ (7.7% ± 2.3 versus 6.5% ± 1.2, resp.; *p* = 0.044), ^51^Cr-EDTA GFR (86 ± 13 versus 101 ± 16 ml/min/1.73m^2^, resp.; *p* = 0.006), age (42 ± 11 versus 35 ± 8 years, resp.; *p* = 0.021), DM duration (25 ± 17 versus 17 ± 5 years, resp.; *p* < 0.001), and H/M_4 (1.52 ± 0.15 versus 1.70 ± 0.22, resp.; *p* = 0.012). In binary logistic regression analysis, both age and HbA_1c_ were independent predictors of ≥2 abnormal CARTs (model's chi-square 30.541, *p* < 0.001).

**Figure 2:**
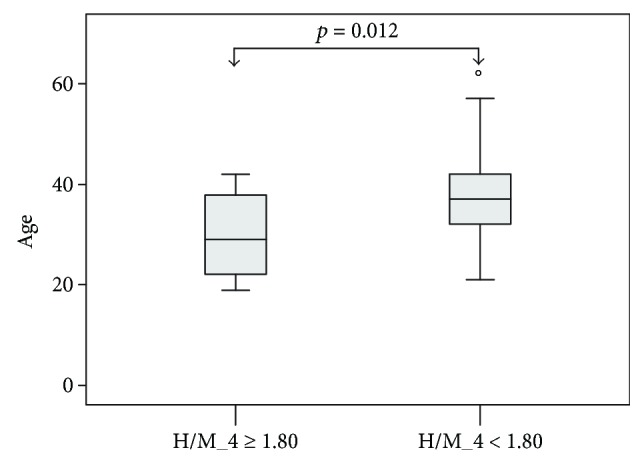
Box plots of age in patients divided according to a cutoff point of ratio of the heart to upper mediastinum count density at 4 hours postinjection (H/M_4) < 1.80 in cardiac ^123^I-metaiodobenzylguanidine scintigraphy.

Patients with H/M_4 < 1.80 (*n* = 37) were older than those with a normal MIBG study (*n* = 12) (38 ± 9 versus 30 ± 9 years, resp.; *p* = 0.012, Figure 2), but there were no differences between the two groups in gender (56.7 versus 66.7% males, resp.; *p* = 0.544), DM duration (20 ± 6 versus 16 ± 6 years, *p* = 0.057) and the number of abnormal CARTs (14/20, 10/16, 5/5, 6/6, and 2/2 abnormal H/M_4 ratios in patients with 0, 1, 2, 3, and 4 abnormal CARTs, resp., *p* = 0.198). In binary logistic regression analysis, only age was found to represent an independent predictor of H/M_4 < 1.80 (model's chi-square 6.959, *p* = 0.008).

In the 37 patients with available HbA_1c_ measurements, patients with H/M_4 < 1.80 did not differ significantly from those with a normal ratio in age (*p* = 0.085), DM duration (*p* = 0.196), HbA_1c_ (*p* = 0.435), ^51^Cr-EDTA GFR (*p* = 0.253), and the number of abnormal CARTs (*p* = 0.186). No significant predictors of H/M_4 < 1.80 were found when variables with a *p* < 0.20 were entered in binary logistic regression analysis.

## 4. Discussion

The present study compared clinical autonomic function testing and MIGB in T1DM patients with no overt complications or other comorbidities. Our findings suggest that clinical tests and MIBG measurements are not closely associated. A threshold of ≥2 abnormal CARTs determines significant changes in sympathetic integrity, as assessed with MIBG, and has high sensitivity but limited specificity to identify CAN. Among clinical variables, DM duration was identified as a significant determinant of ≥2 abnormal CARTs, whereas age was found to represent an independent predictor of abnormal MIBG findings.

The cardiovascular autonomic system plays a pivotal role in the regulation of heart rate, myocardial contractility, and blood pressure. Our group showed that the severity of CAN is associated with the severity of the left ventricular diastolic dysfunction in both patients with T1DM and type 2 DM [29, 30]. Indeed, CAN is a severe complication of DM with potentially deleterious effects on the outcome of these patients [3, 5, 6, 31]. Thus, early recognition of CAN may be crucial in the management of the disease because achieving normoglycemia may delay or prevent the onset of CAN in T1DM [32, 33]. Even though autonomic symptoms present more commonly in T1DM than in T2DM, these symptoms correlate weakly with deficits in early CAN [34]. The so-called Ewing battery of tests has been introduced many years ago to assess cardiovascular autonomic function and remains popular because these tests are noninvasive, simple, safe, reliable, reproducible, and standardized [7]. However, even though these tests are clinically relevant, they principally assess the parasympathetic system.

Myocardial ^123^I-MIBG allows direct visualization and quantification of sympathetic presynaptic neuronal integrity [9]. However, it should be mentioned that uptake measurements are semiquantitative and not an index of absolute neuronal retention. Besides, the delivery of tracers is influenced by myocardial perfusion, and measurements may be influenced by body weight, a number of diseases, and certain medications [2].

The prevalence of CAN detected in our cohort with either CARTs or MIBG is within the range reported in the literature [35]. Our results also demonstrate that MCR abnormalities precede other tests' impairment, whilst MIBG abnormalities occur in the majority of diabetic patients with no abnormal CARTs. Moreover, DM duration and HbA_1c_ were significant predictors of the presence of ≥2 abnormal CARTs. Earlier work has shown inadequate glycemic control to be a leading cause for the development and progression of CAN and duration of the disease to be a significant risk factor [15, 36]. Previous data have also suggested that ^123^I-MIBG scintigraphy may be more sensitive than heart rate variability for detection of CAN in diabetic patients [36, 37]. Furthermore, it has been reported that heart rate variability in deep breathing heralds subclinical CAN and is the most sensitive test to diagnose autonomic dysfunction [2, 35]. Particularly in patients with T1DM, significant correlations were observed between CAN and age, duration of DM, HbA_1c_, retinopathy, microalbuminuria, hypoglycaemia, dyslipidemia, and diastolic blood pressure [14, 38].

Interestingly, since cardiac MIBG scintigraphy addresses the sympathetic autonomic system and CARTs principally assess parasympathetic function, our findings contradict the general notion that parasympathetic (vagal) impairment precedes sympathetic dysfunction during the natural course of CAN in diabetic patients [39]. As denervation occurs in an ascending length-dependent manner, the vagus nerve is usually affected first in CAN, resulting in a relative predominance of the sympathetic tone [35]. At an early stage, this leads to baroreceptor impairment and changes in heart rate variability, but at later stages of the disease, cardiac involvement may become evident. In this respect, however, it has been suggested that MIBG assesses specifically the sympathetic innervation of the heart whereas heart rate variability indices are better markers of systemic autonomic function [35, 39].

The Toronto Consensus Panel required ≥2 abnormal CARTs to define CAN, one abnormal CART for possible CAN, and considered OH with ≥2 abnormal CARTs as indicative of advanced CAN [8]. In the present study, the criterion of ≥2 abnormal CARTs could only discern significant abnormality in cardiac MIBG findings (Table 2). Literally, all patients with ≥2 abnormal CARTs had scintigraphic evidence of sympathetic dysfunction. However, the presence of ≥2 abnormal CARTs had limited specificity, because the majority of T1DM patients with none or only one abnormal CARTs also had sympathetic impairment. Similarly, one abnormal CART was associated with a moderate (63%) likelihood for MIBG abnormality. Regarding the case of postural hypotension (which assesses sympathetic function) plus ≥ 2 abnormal CARTs, in our patients, an abnormal OH invariably was accompanied by one or more additional abnormal CARTs. In these patients, the H/M_4 tended to be low, but no presence of advanced CAN could be confirmed, even after applying a H/M_4 threshold of <1.60.

Age was found to represent an independent predictor of H/M_4 < 1.80. Earlier studies have reported a decrease of cardiac MIBG uptake as a physiological consequence of advancing age [40]. However, more recent reports have challenged this notion and support stability of H/M measurements in patients without coronary heart disease, because changes of numerator parallel those of denominator during ageing [41]. Whatever may be the underlying cause (a consequence of ageing alone or an effect of DM), our data show that advanced age is a marker of CAN in T1DM patients, as demonstrated by a lower cardiac MIBG uptake.

Limitations of the present study include the relatively small sample size, which is particularly important for multivariate analyses. Although a rule does not exist, statistical confidence is substantiated with larger numbers of cases. ^123^I-MIBG measurements were based on an H/M ratio at 4 hours alone, excluding other scintigraphic indices. However, this was preferred for reasons of simplicity in presenting the results, whereas recent data report very similar values between early and late H/M ratios. Notably, although cardiac ^123^I-MIBG testing offers improved sensitivity in detecting autonomic dysfunction and initiating timely therapeutic interventions, it is not known whether this type of assessment is accompanied with improved outcome compared to clinical autonomic testing alone.

Strengths of the present study include the enrollment of well-characterized and young patients with very good metabolic control, early CAN, long duration of DM, without cardiovascular disease, hypertension, or severe microvascular complications.

## 5. Conclusions

In T1DM patients with no complications or cardiovascular risk factors and treated with insulin alone, CAN is common and age is a strong predictor of its presence in cardiac ^123^I-MIBG imaging. Cardiovascular autonomic reflex tests are not closely associated with ^123^I-MIBG measurements, which can detect autonomic dysfunction more efficiently than the former. In comparison to the semiquantitative cardiac MIBG assessment, the recommended threshold of ≥2 abnormal tests is highly sensitive for identifying cardiovascular autonomic dysfunction but has limited specificity and is independently determined by the duration of T1DM. Therefore, MIBG might have to be used in combination with the CARTs for the diagnosis of cardiac autonomic neuropathy in patients with T1DM.

## Figures and Tables

**Table 1:** Patients' characteristics.

Males (%)	59.2
Mean age (years)	36 ± 10
Mean duration of diabetes mellitus (years)	19 ± 6
Mean systolic blood pressure (mmHg)	115 ± 10
Mean diastolic blood pressure (mmHg)	78 ± 8
Mean body mass index (kg/m^2^)	25.2 ± 3.1
Retinopathy (%)	20.4
Microalbuminuria (%)	12.2
Mean HbA_1c_ (%)	6.9 ± 1.5
Mean hematocrit (%)	42.1 ± 1.1
Mean aspartate transaminase (IU/L)	27.3 ± 1.9
Mean alanine transaminase (IU/L)	28.8 ± 1.3
Mean triglycerides (mg/dL)	117.8 ± 20.2
Mean total cholesterol (mg/dL)	159.4 ± 14.5
Mean high-density lipoprotein cholesterol (mg/dL)	48.1 ± 13.3

**Table 2:** Cardiac sympathetic innervation imaging with ^123^I-metaiodobenzylguanidine results as a function of the number of abnormal cardiovascular autonomic reflex tests (CARTs).

	Number of abnormal CARTs					*p*
	0	1	2	3	4	
Patients with H/M_4 < 1.80	14/20 (70%)	10/16 (63%)	5/5 (100%)	6/6 (100%)	2/2 (100%)	0.198
H/M_4	1.68 ± 0.17	1.68 ± 0.26	1.51 ± 0.21	1.50 ± 0.08	1.64 ± 0.20	0.170

H/M_4: ratio of the heart to upper mediastinum count density at 4 hours postinjection.

**Table 3:** Comparison of cardiac sympathetic innervation imaging with ^123^I-metaiodobenzylguanidine (^123^I-MIBG) findings between groups of patients formed according to the number of abnormal cardiovascular autonomic tests (CARTs) versus those with fewer tests than the defined threshold of abnormality.

Number of abnormal CARTs	^123^I-MIBG H/M_4		*p*
	Abnormal group	Normal group	
≥1	1.61 ± 0.22	1.68 ± 0.17	0.234
	(*n* = 29)	(*n* = 20)	
≥2	1.52 ± 0.14	1.68 ± 0.21	0.014
	(*n* = 13)	(*n* = 36)	
≥3	1.53 ± 0.09	1.66 ± 0.21	0.104
	(*n* = 8)	(*n* = 41)	
≥4	1.62 ± 0.11	1.64 ± 0.21	0.916
	(*n* = 2)	(*n* = 47)	
